# Supervised diagnostic classification of cognitive attributes using data augmentation

**DOI:** 10.1371/journal.pone.0296464

**Published:** 2024-01-05

**Authors:** Ji-Young Yoon, Gahgene Gweon, Yun Joo Yoo

**Affiliations:** 1 Department of Mathematics Education, Seoul National University, Seoul, South Korea; 2 Department of Intelligence and Information, Seoul National University, Seoul, South Korea; Zhejiang Normal University, CHINA

## Abstract

Over recent decades, machine learning, an integral subfield of artificial intelligence, has revolutionized diverse sectors, enabling data-driven decisions with minimal human intervention. In particular, the field of educational assessment emerges as a promising area for machine learning applications, where students can be classified and diagnosed using their performance data. The objectives of Diagnostic Classification Models (DCMs), which provide a suite of methods for diagnosing students’ cognitive states in relation to the mastery of necessary cognitive attributes for solving problems in a test, can be effectively addressed through machine learning techniques. However, the challenge lies in the latent nature of cognitive status, which makes it difficult to obtain labels for the training dataset. Consequently, the application of machine learning methods to DCMs often assumes smaller training sets with labels derived either from theoretical considerations or human experts. In this study, the authors propose a supervised diagnostic classification model with data augmentation (SDCM-DA). This method is designed to utilize the augmented data using a data generation model constructed by leveraging the probability of correct responses for each attribute mastery pattern derived from the expert-labeled dataset. To explore the benefits of data augmentation, a simulation study is carried out, contrasting it with classification methods that rely solely on the expert-labeled dataset for training. The findings reveal that utilizing data augmentation with the estimated probabilities of correct responses substantially enhances classification accuracy. This holds true even when the augmentation originates from a small labeled sample with occasional labeling errors, and when the tests contain lower-quality items that may inaccurately measure students’ true cognitive status. Moreover, the study demonstrates that leveraging augmented data for learning can enable the successful classification of students, thereby eliminating the necessity for specifying an underlying response model.

## 1 Introduction

Over the past few decades, an array of machine learning methodologies has emerged, revolutionizing a multitude of sectors including healthcare, finance, engineering, and educational services. As an integral subfield of artificial intelligence, machine learning enables machines to glean knowledge from experience or data. This approach empowers systems to discern patterns, construct predictive models, and make data-driven decisions, all with a minimum requirement for human intervention. Recent advancements in machine learning techniques, including neural networks (NNs) and support vector machines (SVMs), are geared towards addressing classification problems in diverse areas such as computer vision and natural language processing. Both NNs and SVMs predominantly fall into the category of supervised learning, a methodology that educates machine classifiers using labeled data to denote the classes of each observed instance. The advent of large-scale datasets, made accessible through advancements in digital information collection and storage technologies, has significantly enhanced the feasibility of supervised learning [1, 2].

In the field of educational assessment, diagnostic classification models (DCMs), also known as cognitive diagnostic models (CDMs), are developed to pinpoint students’ cognitive proficiencies and deficiencies pertaining to the assessed skills or cognitive attributes [3, 4]. A range of Diagnostic Classification Models (DCMs) has been developed, each predicated on distinct assumptions regarding the *condensation rule* [5]. This rule explicates the interaction between cognitive attributes and how they collectively contribute to the observed item responses. For instance, the Deterministic Inputs, Noisy ‘‘And” Gate (DINA) model [6], which is based on the conjunctive condensation rule, expects that mastery of all required attributes leads to a high probability of answering the item correctly. In contrast, the Deterministic Inputs, Noisy “Or” Gate (DINO) model [7], based on the disjunctive condensation rule, assumes that mastering at least one attribute can result in a high probability of a correct answer. The additive CDM (ACDM) [8], which is based on the additive condensation rule, assumes that each required attribute contributes to the probability of a correct answer.

Model-based estimation techniques, such as Expectation Maximization (EM) and Markov Chain Monte Carlo (MCMC), are commonly employed to fit DCM to educational test data [9]. However, these methods require large sample sizes to achieve stable and reliable parameter estimates [10]. They are also vulnerable to model misspecification, and are sensitive to the quality of test items or the correctness of the Q-matrix specification [11, 12]. Addressing these challenges, several researchers have turned to the methods such as K-means clustering [13] and self-organizing maps [14]. Nonetheless, these unsupervised techniques possess inherent limitations, including their incapacity to directly determine the mastery status of examinees’ cognitive attributes.

In contrast, supervised learning techniques address the diagnostic classification problem head-on by leveraging labeled data of cognitive attribute mastery status. Some researchers have utilized neural networks (NNs) for diagnostic classification by constructing training datasets that are labeled with theoretically expected attribute mastery patterns under attribute hierarchy assumptions or by using information from NNs as priors in MCMC algorithms [15, 16]. Another study proposed the use of cognitive attribute mastery patterns assigned by experts as the labels in the training dataset for SVMs [17]. In the previous studies of DCMs, delving into the use of supervised diagnostic classification models, the size of the training dataset has been limited due to inherent constraints associated with the procurement of labeled data. When working with small training datasets—a common scenario given the challenges of human experts labeling latent mastery statuses—the performance of diagnostic classification tends to be suboptimal. For instance, SVMs trained on expert-labeled datasets reported a pattern-level classification accuracy rate of a mere 0.4, even under conditions with a typical item quality represented by a slip of 0.2 [17].

In the other hand, popular machine learning tasks such as image recognition typically utilize large datasets consisting of thousands of images for training, which contributes to the high performance of the classifiers trained on them [18]. Even when the training dataset size is relatively small, various data augmentation techniques, such as flipping, cropping, rotation, changing color space, and adding noise, have been employed to augment the size and quality of the training dataset, aiming to build more accurate classifiers [19]. Recently, augmentation methods generating synthetic data that closely resembles the original data distribution using generative models have gained popularity, moving beyond merely transforming the original data [20, 21].

Integrating data augmentation with supervised diagnostic classification models could potentially mitigate the challenges posed by small training datasets while retaining the advantages that model-free supervised diagnostic classification hold over DCMs with model-based estimation. However, to the best of our knowledge, no study has yet applied data augmentation to supervised diagnostic classification models. While a handful of studies have implemented data augmentation in model-based approach for DCMs [22–24], the augmentation techniques borrowed in these studies are rooted in traditional statistical learning. These methods primarily aim to compute posterior distributions and increase computational speed in likelihood-based model estimation [25]. To successfully incorporate data augmentation into SDCMs, it is imperative to devise augmentation strategies that harness the distinct features of assessment data without any distributional assumptions.

Building on these ideas, this paper presents a novel approach for DCM, the Supervised Diagnostic Classification Model with Data Augmentation (SDCM-DA). This methodology introduces a technique to augment the labeled training dataset, upon which a machine classifier is then trained. The resulting augmented training dataset effectively embodies real-world data in tandem with an expert-labeled dataset, aspiring to boost the performance of classification within the supervised learning paradigm in DCMs. Moreover, the proposed SDCM-DA method is model-independent, and can become a promising alternative to model-based estimation in DCM, which requires a large sample size to obtain stable and reliable estimates and sometimes suffers from model misspecification or misfit [26].

The structure of this paper is as follows. In the succeeding section, we offer a succinct overview of two prominent supervised diagnostic classification models: Neural Networks (NNs) and Support Vector Machines (SVMs), in addition to detailing the data augmentation methods currently in use for various machine learning challenges. Subsequently, we introduce a novel approach, the Supervised Diagnostic Classification Model with Data Augmentation (SDCM-DA), which employs supervised learning for the diagnostic classification of cognitive attribute mastery by augmenting expert-labeled data of limited size. In the subsequent section, we undertake a simulation study to appraise and compare the efficacy of machine learning-based diagnostic classification employing NNs and SVMs. Finally, the discussion section summarizes our key findings, enumerates challenges, and proposes avenues for future research.

## 2 Background

### 2.1 Diagnostic classification models

A myriad of Diagnostic Classification Models (DCMs) have been developed in accordance with diverse condensation rules. In this section, we delve into three salient models—DINA, DINO, and ACDM. Each of these models embody distinct condensation rules, conjunctive, disjunctive, and additive, respectively.

The deterministic-input, noisy-and-gate (DINA) model [6] is a non-compensatory model that features a conjunctive condensation rule. This rule implies that the mastery of all required attributes is imperative for an examinee to respond correctly to an item. The variable *η*_*jc*_ is used to indicate whether an examinee with an attribute mastery pattern of ***α***_*c*_ has successfully mastered all required attributes for item *j*. It is mathematically represented as follows:

$$\eta_{jc}=\prod_{k=1}^{K}\alpha_{ck}^{q_{jk}},$$

where the indicator variable *α*_*ck*_ denotes whether an examinee with an attribute mastery pattern of ***α***_*c*_ has mastered the required attribute *k* (*α*_*ck*_ = 1) or not (*α*_*ck*_ = 0), *q*_*kj*_ indicates whether item *j* measures the cognitive attribute *k* (*q*_*jk*_ = 1) or not (*q*_*jk*_ = 0), and *K* is the number of attributes.

The DINA model also considers ‘slip’ and ‘guess’ parameters. It contemplates that examinees who have mastered all required attributes (*η*_*jc*_ = 1) may ‘slip’ and answer item *j* incorrectly, and those who have not mastered at least one of the required attributes (*η*_*jc*_ = 0) may ‘guess’ and answer correctly. The parameters slip *s*_*j*_ and guess *g*_*j*_ capture these instances, respectively. Consequently, under DINA model, the probability that an examinee with an attribute mastery pattern of ***α***_*c*_ answers item *j* correctly can be written as follows:

$$P\left(X_{j}=1|\alpha_{c}\right)={\left(1-s_{j}\right)}^{\eta_{jc}}{g_{j}}^{1-\eta_{jc}}$$

where *X*_*j*_ indicates whether an examinee with the attribute mastery ***α***_*c*_ answers item *j* correctly.

The deterministic input, noisy “or” gate (DINO) model [7] adopts a disjunctive condensation rule, implying that mastery of at least one of the required attributes enables an examinee to answer an item correctly. The variable *ω*_*jc*_ indicates whether an examinee with an attribute mastery pattern of ***α***_*c*_ has successfully mastered at least one required attribute for item *j*, as follows:

$$\omega_{jc}=1-\prod_{k=1}^{K}{\left(1-\alpha_{ck}\right)}^{q_{jk}}.$$

The DINO model also accounts for the possibilities of ‘slip’ and ‘guess’. It considers that examinees who have mastered at least one required attribute (*ω*_*jc*_ = 1) may ‘slip’ and answer incorrectly to item *j*, and those who have not mastered any attribute (*ω*_*jc*_ = 0) may ‘guess’ and answer correctly. The parameters slip *s*_*j*_ and guess *g*_*j*_ represent these instances, respectively. Considering these factors, under DINO model, the probability that an examinee with an attribute mastery pattern of ***α***_*c*_ correctly answers item *j* can be articulated as follows:

$$P\left(X_{j}=1|\alpha_{c}\right)={\left(1-s_{j}\right)}^{\omega_{jc}}{g_{j}}^{1-\omega_{jc}}.$$

The Additive Cognitive Diagnosis Model (ACDM) proposes that the probability of a correct response to an item varies depending on the mastery of the required attributes [8]. For notational convenience but without loss of generality, let the first $K_{j}^{*}$ attributes be required for item *j*. Within the ACDM, the item response function that delineates the probability of an examinee with a cognitive pattern ***α***_*c*_ correctly answering item *j* is formulated as follows:

$$P\left(X_{j}=1|\alpha_{cj}^{*}\right)=\delta_{j0}+\sum_{k=1}^{K_{j}^{*}}\delta_{jk}\alpha_{ck},$$

where $K_{j}^{*}=\sum_{k=1}^{K}q_{kj}$ represents the number of required attributes for item *j*, and $\alpha_{cj}^{*}$ is the reduced attribute vector of ***α***_*c*_, whose elements consist of only the required attributes for item *j* [8]. Then, *δ*_*j*0_ signifies the probability of correctly answering item *j* when none of the required attributes have been mastered, and the variable *δ*_*jk*_ represents the effect attributed to the *k*-th cognitive attribute related to item *j*, referred to as the main effect of the *k*-th cognitive attribute.

As inferred from the form of the item response function, the ACDM postulates that mastering attribute *k* enhances the probability of a correct response to item *j* by *δ*_*jk*_. This reflects the additive nature of this model, distinct from the conjunction and disjunction rules of the DINA and DINO models, respectively.

### 2.2 Supervised diagnostic classification model

Model-based estimation has been commonly used in the previous studies on DCMs for inferring cognitive patterns [6–8]. However, cognitive diagnosis can also be considered as a classification problem, where students are classified into cognitive mastery patterns based on their performance in solving problems that necessitate the application of specific cognitive skills. This opens up the possibility of applying machine learning methods to Diagnostic Classification Models (DCMs), although such studies are limited in number.

In the sections that follow, we offer a concise overview of two machine learning methods that can be applied to DCM, Neural Networks (NNs) and Support Vector Machines (SVMs), accompanied by a review of methods in DCMs that employ these specific supervised learning techniques. We term this line of method the Supervised Diagnostic Classification Model (SDCM).

#### 2.2.1 Neural networks

The feed-forward neural network, also known as the multilayer perceptron, is the most successful and frequently used model of NNs [27]. A simple version of NN comprises three layers: the input layer, the output layer, and a hidden layer located between the input and output layers. Each layer consists of nodes denoting variables, and directed edges connect the nodes in a layer to those in the subsequent layer, starting from the input layer. A NN establishes relationships between the input and output layers through a set of linking functions connecting consecutive layers.

A NN with one hidden layer including *H* nodes can be modeled as follows: Suppose that the input and output layers have *J* and *K* nodes, respectively. For a given input data point ***x* = (***x*_1_, *x*_2_, …, *x*_*J*_) in the training dataset, the *k*-th output variable *y*_*k*_ of the network’s output vector ***y* = (***y*_1_, *y*_2_, …, *y*_*K*_) is computed as follows:

$$y_{k}\left(x,\;w\right)=\sigma \left(\sum_{l=1}^{H}w_{kl}^{\left(2\right)}h\left(\sum_{j=1}^{J}w_{lj}^{\left(1\right)}x_{j}+w_{0}^{\left(1\right)}\right)+w_{0}^{\left(2\right)}\right),$$

where $w_{lj}^{\left(1\right)}$ denotes the weight connecting node *j* of the input layer to node *l* of the hidden layer, $w_{kl}^{\left(2\right)}$ denotes the weight connecting node *l* of the hidden layer to node *k* of the output layer, $w_{0}^{\left(1\right)}$ denotes a bias (intercept) of the input layer, $w_{0}^{\left(2\right)}$ denotes a bias (intercept) of the hidden layer, and ***w*** is the set of weights $w_{lj}^{\left(1\right)}$ and $w_{kl}^{\left(2\right)}$, for *j* = 1, …, *J*, *l* = 1, …, *H* and *k* = 1, …, *K*. In addition, *h*(∙) and *σ*(∙) are activation functions in the hidden and output layers, respectively. For the activation function, nonlinear functions such as the sigmoid function, *tanh* function, and ReLU function are often used [28]. Specifically, by using the sigmoid function for the second activation function *σ*, *y*_*k*_ has a value between 0 and 1, which can be interpreted as the probability of the *k*^*th*^ variable of the output layer to be 1. Weight values that minimize the errors between the actual and predicted values are obtained by a backpropagation algorithm using a training dataset of *N* observations, each of which comprises of the input vector and the vector of corresponding label (output vector).

To perform diagnostic classification using neural networks (NNs), we train the network by using the item response vectors as inputs, which represent the binary responses of students indicating correct and incorrect answers, and providing the pre-labeled attribute profiles as outputs. The trained neural network (NN) can diagnose attribute profiles for new student responses on the same items used in the training dataset. The output values for each node corresponding to each attribute are transformed values ranging from 0 to 1 using the sigmoid function for *σ*(∙), which can be interpreted as the probability of mastering the attribute. The examinee can be classified into an attribute profile by comparing the output values with a cutoff point of 0.5, for attribute-wise diagnosis, or by comparing them with possible attribute patterns using similarity measures, such as the mean-squared difference for pattern-wise diagnosis [14, 15].

The previous studies [15, 16] that utilized NNs as supervised diagnostic classification models used the theoretically determined dataset as their training set. This dataset consists of all possible attribute mastery patterns as labels and expected response patterns as inputs corresponding to each possible attribute pattern. The construction of this dataset is based only on the response model of DCMs and Q-matrix, assuming that there are no errors in item responses. The theoretically determined training dataset has the advantage of being able to diagnose examinees without the empirically obtained training dataset, so that, regardless of the size of the examinees, it can be easily applied. However, this dataset is peculiar in both the DCM framework and machine learning field, as it disregards response errors such as slip and guess that are considered fundamental parameters of DCMs [6, 7], and it only contains one theoretically determined instance per class, making it an atypical training dataset. Additionally, this dataset heavily relies on assumed response models such as DINA and DINO, making performance of the trained model significantly impacted in case of model misspecification.

#### 2.2.2 Support vector machines

Support Vector Machines (SVMs) are powerful and versatile supervised machine learning models that are widely used for classification, regression, and novelty detection [29, 30]. SVMs can efficiently classify data into two groups by finding the optimal hyperplane that not only separates the two groups, but also has the largest classification margin. Denote the training samples as (***x***_1_, *y*_1_), (***x***_2_, *y*_2_), …, (***x***_*N*_, *y*_*N*_), where the variable *y*_*i*_ indicates the group to which the instance ***x***_*i*_ belongs by 1 or −1. The optimal hyperplane ***w*** ∙ ***x*** + *b* = 0 that separates two groups can be determined by finding the parameters ***w*** and *b* satisfying the following conditions:

$$y_{i}\left(w∙x_{i}+b\right)\geq 1,$$

$$\underset{w,b}{\text{argmin}}\;{\left\|w\right\|}^{2},$$

for *i* = 1, …, *N*. These constraints assume that the training data points are linearly separable, which means that at least one hyperplane separates them into two groups. However, in practice, when class-conditional distributions overlap, the two groups cannot be completely separated by any hyperplane. In such cases, the soft-margin approach is used to allows data points to remain on the wrong side of the margin boundary. The slack variable *ξ*_*I*_ ≥ 0 is introduced to impose the penalty as the distance from the margin boundary to the instances classified on the wrong side. The constraints for the soft-margin SVM are then modified as:

$$y_{i}\left(w∙x_{i}+b\right)\geq 1-\xi_{i},$$

$${\text{argmin}}_{w,b}\;{\left\|w\right\|}^{2}+C\sum_{i=1}^{N}\xi_{i},$$

for *i* = 1, …, *N*, where parameter *C* > 0 controls the trade-off between the margin and slack variable penalty. The soft-margin approach allows SVMs to handle non-linearly separable data points and is widely used in practice.

SVMs, being a binary classification model, can be used to diagnose students on their cognitive attributes. In the case where the test consists of *J* items measuring *K* attributes, the student’s item response data is represented as data points in a *J*-dimensional space. *K* SVMs are required to diagnose the student’s cognitive profile for each of the *K* attributes. The input for each SVM is a reduced-dimensional response vector of length J* for the J* items that measure the corresponding attribute, based on the Q-matrix. Then, the SVM model classifies the data points into binary classes of attribute mastery (1) or non-mastery (0). Note that the above description for SVMs assumes a linear kernel, but it can also be applied using a non-linear kernel.

Liu and Cheng [17] utilized SVM as a supervised diagnostic classification model, training it on an expert-labeled dataset. The dataset included students’ response patterns and mastery profiles, obtained through expert or teacher labeling. The labeling process of the training samples could incorporate auxiliary data either explicitly or implicitly. However, the expert labeling process required significant resources, making it difficult to obtain large-scale training datasets. Nevertheless, Liu and Cheng [17] observed that SVMs trained on expert-labeled datasets of size 30 successfully addressed the challenge of limited training dataset by achieving a 90% accuracy rate, despite a 10% labeling error rate. It is important to note, though, that expert-labeled training datasets still have their limitations. Subjectivity in evaluations can introduce errors in the labeling results, and the small size of expert-labeled datasets may not provide a comprehensive representation of the entire population, potentially resulting in overfitting.

In this article, we propose a straightforward and effective data augmentation approach utilizing the expert-labeled dataset to generate abundant training dataset. This method aims to overcome the aforementioned limitations of the expert-labeled dataset and construct more robust supervised classifiers.

### 2.3 Data augmentation for training process

Data augmentation is a crucial aspect of training deep learning models, as it is widely recognized that larger datasets lead to improved model performance [31, 32]. The performance of a classifier heavily relies on a substantial labeled dataset. However, procuring such a labeled dataset can be time-consuming, costly, and challenging. To address this, researchers have proposed various data augmentation methods to increase the size and diversity of dataset. Initially designed to reduce the time and cost associated with data collection and enhance the size and diversity of datasets, data augmentation methods have evolved to serve various purposes, from resolving class imbalance to addressing privacy issues. Data augmentation methods can be broadly categorized into a basic manipulation approach, also referred to as semantically invariant transformation, and a deep learning approach, which utilizes deep learning to learn the data generating distribution.

The basic manipulation approach employs modifications to existing data to generate augmented data suited for specific purposes. For instance, image data can be augmented through basic manipulations like geometric transformations, color adjustments, random erasing, kernel filters, and image mixing [33–37]. These augmentation processes can enhance the diversity and mitigate biases of the training dataset. Text data can be augmented at character, word, sentence, or document levels [38] using techniques such as random character modification [39, 40], synonym replacement, context-based word generation via masked language models [41–44], or back translations [45].

In contrast, the deep learning approach employs generative models to create synthetic data closely resembling the original data distribution. The approach includes methods based on models like Variational Autoencoders (VAEs), Generative Adversarial Networks (GANs), and diffusion models, which can be applied to augment various data types like images, text, or voice. VAEs [20] map input data to a latent space and reconstruct it, while GANs [21, 46] utilize generators trained to distinguish between real and synthetic data. Diffusion models [47] learn the data distribution by iteratively adding noise and reconstructing the original data.

To our knowledge, data augmentation has not been previously applied in the context of supervised diagnostic classification using students’ performance data. Unlike image or text data, assessment data, composed of item response patterns and labels of attribute mastery, does not possess an obvious invariant structure, making such a basic manipulation approach inapplicable. Additionally, the deep learning approach often requires significant amounts of data to train models for data augmentation [48], posing challenges to its application in supervised diagnostic classification. In fact, many studies using deep learning approaches tend to rely on large amounts of data [48, 49] or use pre-trained models [50, 51] to train the model for augmentation. Therefore, we present a simple yet efficient data augmentation method specifically designed for item response data and apply it in the process of supervised diagnostic classification.

## 3 The proposed SDCM-DA method

We present a novel Supervised Diagnostic Classification Model using Data Augmentation (SDCM-DA). This method leverages supervised learning combined with data augmentation techniques to bolster model performance. The process involves two crucial steps. In the initial stage, we glean the probabilities of correct answers from an expert-labeled dataset, establishing the bedrock for the forthcoming procedures. Subsequently, we leverage these probabilities to generate an ideally large dataset, thereby facilitating efficient training of the SDCM-DA.

The first step of extracting the probabilities of correct answer is detailed below. The correct answer probabilities for each reduced attribute mastery pattern are extracted only based on Q-matrix. These probabilities are extracted from the intrinsically small expert-labeled dataset, which consists of item responses and labels diagnosed by experts. Suppose a test with *J* items measures *K* binary attributes. For notational simplicity, let us assume that the first $K_{j}^{*}$ attributes are required for item *j*, and $\alpha_{l}^{*}=\left(\alpha_{l1},\;\ldots ,\;\alpha_{lK_{j}^{*}}\right)$ is the *l*th reduced attribute mastery pattern, where *α*_*lk*_ indicates the mastery of attribute *k* for $l=1,\;\ldots ,2^{K_{j}^{*}}$. Let *X*_*j*_ be the response to item *j*, denoted as 1 for a correct response and 0 for an incorrect response. The probability of correctly answering item *j* with the reduced attribute pattern $\alpha_{l}^{*}$ is denoted by $p_{lj}=P\left(X_{j}=1|\alpha_{l}^{*}\right)$. This can be estimated from the expert-labeled dataset as the ratio of examinees who correctly answered the item with the reduced labeled attribute pattern $\alpha_{l}^{*}$ to the total number of examinees with the reduced attribute pattern $\alpha_{l}^{*}$. It can be formulated as follows:

$${\hat{p}}_{lj}=\frac{R_{lj}}{I_{lj}}$$

where *I*_*lj*_ is the number of examinees with the reduced mastery pattern $\alpha_{l}^{*}$ for item *j*, and *R*_*lj*_ is the number of those examinees who answered the item *j* correctly among *I*_*lj*_ examinees having the pattern $\alpha_{l}^{*}$. For example, for item *j* that requires two attributes, the probabilities of correct answer are calculated for four reduced mastery patterns 00, 10, 01, 11. With the expert-labeled dataset, $P\left(X_{j}=1|\alpha_{l}^{*}=(00)\right)$ is calculated as the ratio of the number of examinees possessing a reduced attribute pattern of (00) and answered item *j* correctly to the total number of examinees possessing a reduced attribute pattern of (00). Analogously, $P\left(X_{j}=1|\alpha_{l}^{*}=(10)\right),\;P\left(X_{j}=1|\alpha_{l}^{*}=(01)\right)$, and $P\left(X_{j}=1|\alpha_{l}^{*}=(11)\right)$ are derived in a congruent manner. If an expert-labeled dataset lacks an examinee with a specific reduced attribute pattern $\alpha_{l}^{*}$, making the corresponding probability incalculable, then the missing probability is randomly sampled from following uniform distribution:

$$P\left(X_{j}=1|\alpha_{l}^{*}\right)\sim U\left(\frac{t_{lj}}{K^{*}+1},\;\frac{t_{lj}+1}{K^{*}+1}\right),$$

where *t*_*lj*_ is the number of attributes taken as mastered in the pattern $\alpha_{l}^{*}$ for $l=1,\;\ldots ,2^{K_{j}^{*}}$. This distribution is based on the assumption that “the greater the number of cognitive attributes mastered, the higher the probability of answering correctly.” Such an additive assumption is also used as a setting in simulation studies for the generalized DINA(G-DINA) model, one of the most generalized models of DCMs [8, 52].

In the subsequent step of constructing an augmented dataset, we generate a dataset comprising *N* pairs of attribute mastery patterns and item response patterns. *N* attribute mastery patterns are randomly selected with replacement from the set of all possible attribute patterns. Utilizing the previously extracted correct response probabilities and the Q-matrix, we generate item response patterns corresponding to each attribute pattern. To clarify, the item response *X*_*j*_ for item *j* given the attribute mastery pattern ***α*** is derived using a Bernoulli distribution with a probability $P\left(X_{j}=1|\alpha_{l}^{*}\right)$, when the reduced attribute pattern for item *j* pertaining to ***α*** is denoted as $\alpha_{l}^{*}$. By employing this method, *N* virtual item response patterns are generated for *N* attribute mastery patterns. These *N* pairs of attribute mastery patterns and item response patterns constitute the augmented training dataset. This approach enables the construction of an extensive training dataset independent of any specific DCM and reflects the item response errors estimated from the expert-labeled data.

Finally, a supervised model can be trained on this augmented dataset. In this study, we try two of popular supervised models: NNs and SVMs. NNs are considered as the fundamental model in the field of deep learning, and SVMs are one of the most popular types of machine learning and are known for their ability to generalize well with a limited amount of training dataset [53]. We also chose these two models, NNs and SVMs, because previous studies [15–17] have also explored the application of both NNs and SVMs as supervised diagnostic classification models using either very small theoretically constructed data or expert-labeled data of small size.

## 4 Simulation study

A simulation study is designed to evaluate the performance enhancement of supervised diagnostic classification using the proposed data augmentation. The cognitive diagnosis results obtained by SDCM-DA are contrasted with those obtained from supervised diagnostic classification that is trained solely on the expert-labeled dataset without any data augmentation. Furthermore, we compare the performance of SDCM-DA with those of model-based estimation in DCMs with correct or incorrect assumption of underlying item response model: DINA [6], DINO [7] or ACDM [8].

The supervised machine learning methods used in this study are NNs and SVMs, which have been utilized in previous diagnostic classification studies [15–17]. The simulation study is executed employing proprietary R codes for data generation, item parameter estimation, and performance evaluation. Additionally, the ‘nnet’ package is utilized for implementing NNs [54], the ‘e1071’ package for SVMs [55], and the ‘CDM’ package for model-based estimation of DINA, ACDM, and DINO models [56].

### 4.1 Design

The simulation study takes into consideration multiple factors, including (a) the item response generation model with three distinct condensation rules, (b) the level of item quality reflected in item parameters, also with three unique settings, (c) the sample size of the expert-labeled dataset, comprising six settings, (d) the size of the augmented training dataset, with six potential settings, and (e) the expert-labeling error rate, which also has six different settings.

The specifics of the simulation setup are outlined as follows. Consistent with Liu and Cheng’s study [17], the number of items and attributes are consistently set at *J* = 30 and *K* = 6, respectively. The Q-matrix of each test scenario consists of 1-attribute items and 2-attribute items, with all attributes evenly measured by eight items (Table A in S1 Appendix).

Three item response models–DINA, ACDM, and DINO—are used to generate item responses given a specific cognitive attribute mastery profile. These item response models are formulated using item parameters that are determined corresponding to the level of item quality. The item response models, expressed using the parameters specific to each model, are summarized in Table 1.

**Table 1:** Item response probabilities of DINA, DINO and ACDM models.

		DINA	DINO	ACDM(re)
1-attribute item	$P\left(X_{j}=1|\alpha_{j}^{*}=0\right)$	*g*_*j*_	*g*_*j*_	*g*_*j*_
	$P\left(X_{j}=1|\alpha_{j}^{*}=1\right)$	1 − *s*_*j*_	1 − *s*_*j*_	1 − *s*_*j*_
2-attribute item	$P\left(X_{j}=1|\alpha_{j}^{*}=\left(0,\;0\right)\right)$	*g*_*j*_	*g*_*j*_	*g*_*j*_
	$P\left(X_{j}=1|\alpha_{j}^{*}=\left(1,\;0\right)\right)$	*g*_*j*_	1 − *s*_*j*_	*m*_*j*_
	$P\left(X_{j}=1|\alpha_{j}^{*}=\left(0,\;1\right)\right)$	*g*_*j*_	1 − *s*_*j*_	*g*_*j*_ + (1 − *s*_*j*_) − *m*_*j*_
	$P\left(X_{j}=1|\alpha_{j}^{*}=\left(1,\;1\right)\right)$	1 − *s*_*j*_	1 − *s*_*j*_	1 − *s*_*j*_

*Note*. ACDM(re): reparameterization of ACDM.

For each item response generation model, item parameters are set to facilitate random sampling for a specified item quality condition. The item response function for an item requiring a single attribute can be represented using the slip *s*_*j*_ and guess *g*_*j*_ parameters for all three models. For ACDM, the parameters *δ*_*j*0_ and *δ*_*j*1_ can be reparameterized as following:

$$P\left(X_{j}=1|\alpha_{j}^{*}=0\right)=\delta_{j0}=g_{j},$$

and

$$P\left(X_{j}=1|\alpha_{j}^{*}=1\right)=\delta_{j0}+\delta_{j1}=1-s_{j}.$$

For items requiring two attributes, the item response function for ACDM can be reparameterized using an additional parameter, *m*_*j*_. The relationships between the original parameters of *δ*_*j*0_, *δ*_*j*1_ and *δ*_*j*2_, and the new parameters of *s*_*j*_, *g*_*j*_, and *m*_*j*_ are as follows:

$$P\left(X_{j}=1|\alpha_{j}^{*}=\left(0,\;0\right)\right)=\delta_{j0}=g_{j},$$

$$P\left(X_{j}=1|\alpha_{j}^{*}=\left(1,\;0\right)\right)=\delta_{j0}+\delta_{j1}=m_{j},$$

$$P\left(X_{j}=1|\alpha_{j}^{*}=\left(0,\;1\right)\right)=\delta_{j0}+\delta_{j2}=g_{j}+\left(1-s_{j}\right)-m_{j},$$

and

$$P\left(X_{j}=1|\alpha_{j}^{*}=\left(1,\;1\right)\right)=\delta_{j0}+\delta_{j1}+\delta_{j2}=1-s_{j}.$$

The item parameters *s*_*j*_ and *g*_*j*_ for item *j* are determined based on the item quality level such that they are sampled from a uniform distribution: *U*(0, 0.1), *U*(0.1, 0.2), and *U*(0.2, 0.3), respectively for good, medium and bad. In addition, the additional parameter *m*_*j*_, used in the ACDM, is also randomly assigned based on the item quality level. Considering the additive characteristic of ACDM, the item parameter *m*_*j*_ for item *j* is sampled from a uniform distribution: *U*(0.1, 0.9), *U*(0.2, 0.8) and *U*(0.3, 0.7) respectively for good, medium and bad level.

In this study, the sample size *M* of the expert-labeled training dataset is set to 10, 20, 30, 50, 100, or 150. These numbers are consistent with a previous study which identified the advantages of SVMs in small-sample scenarios [17]. For each sample size *M*, the number of examinees who master each attribute is constrained to be within the range $\left[\frac{M}{5},\;\frac{4M}{5}\right]$ to avoid the imbalance problem [57]. The size *N* of augmented dataset is set to 300, 500, 1,000, 3,000, 5,000, or 10,000.

The effects of different error rates in labeling are examined in the simulation study. The error rate is defined as the rate at which examinees are diagnosed with a mastery pattern that is not an actual attribute mastery pattern. To comprehensively examine the error rate factor, 6 levels of error rates are considered: 0, 0.1, 0.2, 0.3, 0.4 and 0.5.

The simulation conditions for cognitive diagnostic classification through supervised learning, as previously detailed, are summarized in Table 2.

**Table 2:** Simulation conditions for supervised diagnostic classification.

factor	conditions	
Item response model	DINA, ACDM, DINO	
Item quality	Good	: *s*_*j*_, *g*_*j*_ ~ *U*(0, 0.1)
	medium	: *s*_*j*_, *g*_*j*_ ~ *U*(0.1, 0.2)
	bad	: *s*_*j*_, *g*_*j*_ ~ *U*(0.2, 0.3)
Size of expert-labeled dataset	10, 20, 30, 50, 100, 150	
Size of augmented dataset	300, 500, 1,000, 3,000, 5,000, 10,000	
Expert-labeling error rate	0, 0.1, 0.2, 0.3, 0.4, 0.5	

Note that the results of cognitive diagnosis by model-based estimation are obtained for each of nine pairs of ‘true model vs. assumed model’—all possible combinations, comprising three instances of accurate model specification and six instances of model misspecification.

### 4.2 Simulation process

Under each unique combination of factors in each simulation, expert-labeled training datasets, augmented training datasets, and test datasets are generated. The examinees for the expert-labeled datasets across various sample sizes *M* are produced through random sampling of all possible attribute patterns. A similar method is employed to generate a total of 5,000 examinees for the test datasets. Item response data is then formed, using the Q-matrix and the sampled item parameters across three different item quality levels, predicated on the underlying DINA, ACDM, or DINO models. For expert-labeled datasets, labels are also generated at varying error rates. When the error rate is zero, labels for each attribute mastery status are assigned according to the initially sampled attribute pattern of that examinee without adding additional noise factor. With a nonzero error rate, while labels from the zero-error scenario are largely retained, a fraction corresponding to the error rate is substituted with incorrect attribute mastery patterns to depict mis-diagnosed labels. Augmented training datasets of size *N* are generated using the probabilities of correct responses for each attribute mastery pattern, drawn from each of the expert-labeled datasets with varying sizes and error rates.

Utilizing the NNs and SVMs trained on these generated training datasets (both expert and augmented) under each condition of item quality, the size of expert-labeled data, and the labeling error rate, the examinees of the test dataset are diagnosed, with the diagnostic accuracy subsequently evaluated. Meanwhile, as a comparative benchmark, the model-based estimation is also examined. This is achieved by training the model-specified DCM with the expert-labeled dataset and then evaluating its performance with the test dataset. The test dataset used to evaluate the performance of DINA, ACDM, and DINO is the same as the one used for SCDM-DA.

This entire process is repeated 50 times for each simulation scenario to ensure the stability of the results. All the simulation findings are then validated through an independent replication of the entire process.

### 4.3 Machine settings and cross-validation

In line with a previous study [15], the NN model was configured with a single hidden layer comprising nine hidden nodes. For the SVM, the tuning parameter *C* for the soft-margin linear-kernel SVM was determined via 10-fold cross-validation for two kinds of training datasets: the expert-labeled dataset and the augmented dataset produced using the SDCM-DA method. Preliminary findings revealed differing optimal *C* values for each type of training dataset, leading us to ascertain distinct *C* values for each. In the case of the expert-labeled training dataset, cross-validation outcomes indicated that *C* = 0.1 and *C* = 0.01 held similar promise. Consequently, aligning with Liu and Cheng’s study [17], we selected *C* = 0.1 as the optimal value for the expert-labeled training dataset. For the augmented training dataset, our investigation determined that *C* = 0.001 provided the best results under the majority of conditions.

### 4.4 Accuracy measures

To evaluate the impact of SDCM-DA, we used two measures of accuracy of attribute profile diagnosis: the Pattern-wise Agreement Rate (PAR; or the attribute profile classification rate) and the Attribute-wise Agreement Rate (AAR; or the marginal attribute classification rate) as used in previous studies [17, 58–60]. The PAR is defined as the proportion of attribute patterns accurately classified across all examinees: $PAR=\sum_{j=1}^{N_{T}}\frac{I\left[\alpha_{c}^{j}=\alpha_{d}^{j}\right]}{N_{T}},$ where $\alpha_{c}^{j}$ is the true profile of the *j*^*th*^ examinee and $\alpha_{d}^{j}$ is the diagnosed profile of the *j*^*th*^ examinee for *N*_*T*_ examinees of test dataset. The AAR is the proportion of times a single attribute is classified correctly across all attributes and examinees: $AAR=\sum_{j=1}^{N_{T}}\sum_{k=1}^{K}\frac{I\left[\alpha_{ck}^{j}=\alpha_{dk}^{j}\right]}{N_{T}K}$ where $\alpha_{ck}^{j}$ and $\alpha_{dk}^{j}$ are the *j*^*th*^ examinee’s true and diagnosed profiles of *k*^*th*^ attribute, respectively, for *N*_*T*_ examinees of test dataset. Each measure is averaged over 50 replications for each simulation condition.

### 4.5 Results

#### 4.5.1 Effectiveness of SDCM-DA

**Fig 1:**
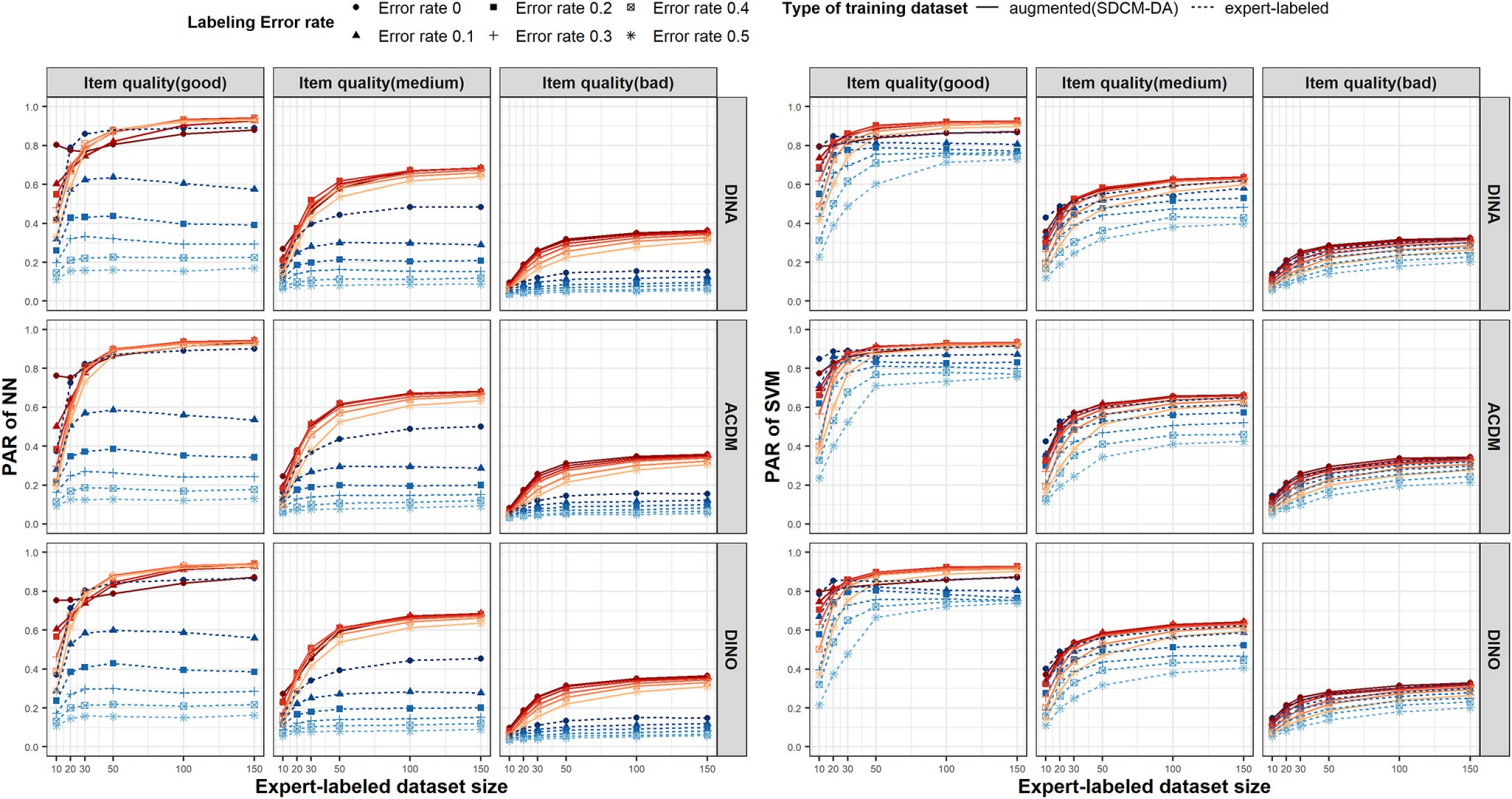
Pattern-wise agreement rates of NN (left) and SVM (right). The group of red solid lines represents the performance of models trained on the augmented training dataset, and the group of blue dashed lines represents the performance of models trained on the expert-labeled training dataset. The degree of expert-labeling error rate is symbolized by the vividness of the colored lines, with a more vivid color indicating a smaller error rate. The subgraphs in the first row illustrate the case when the item response generation model is DINA, while the second and third rows represent the cases for ACDM and DINO, respectively.

In order to assess the effectiveness of the SDCM-DA method, we compare the classification accuracies, measured by the PARs and AARs, of two supervised machine learning models (NN and SVM) trained using the augmented dataset, created through our proposed method, and the original expert-labeled dataset. This comparison spans various simulation conditions, considering different item quality settings, labeling error rates, and sizes of the expert-labeled dataset. Fig 1 displays the PARs for NN (shown on the left) and SVM (shown on the right) when the augmented dataset size is fixed at N = 10,000. An additional figure displaying the average accuracy of AAR is presented in the supporting information (Fig B in S1 Appendix).

Overall, the accuracy of both NN and SVM has seen an enhancement through the application of the SDCM-DA method, in comparison to the results obtained when solely using the expert-labeled dataset for training. For example, when using the SDCM-DA augmented dataset under the DINA model, the PARs of the NN demonstrate an improvement of 49%–192% compared to when the model was trained solely with the expert-labeled dataset, even with a labeling error rate of 0.1 and bad-item quality from the expert labeling, as evidenced in Fig 1. As the labeling error rate in expert labeling increased, the degree of improvement achieved by adopting the SDCM-DA method became even more pronounced. With the medium-item quality and labeling error rate of 0.4, the PAR (AAR) has improved from 0.11 (0.69) to 0.64 (0.92) when the size of expert-labeled dataset is 100. For brevity, we primarily described the results for DINA model; the exact numerical values for ACDM or DINO models, which bear substantial similarity to DINA, can be found in the result table of S2 Appendix.

#### 4.5.2 Size of augmented dataset

**Fig 2:**
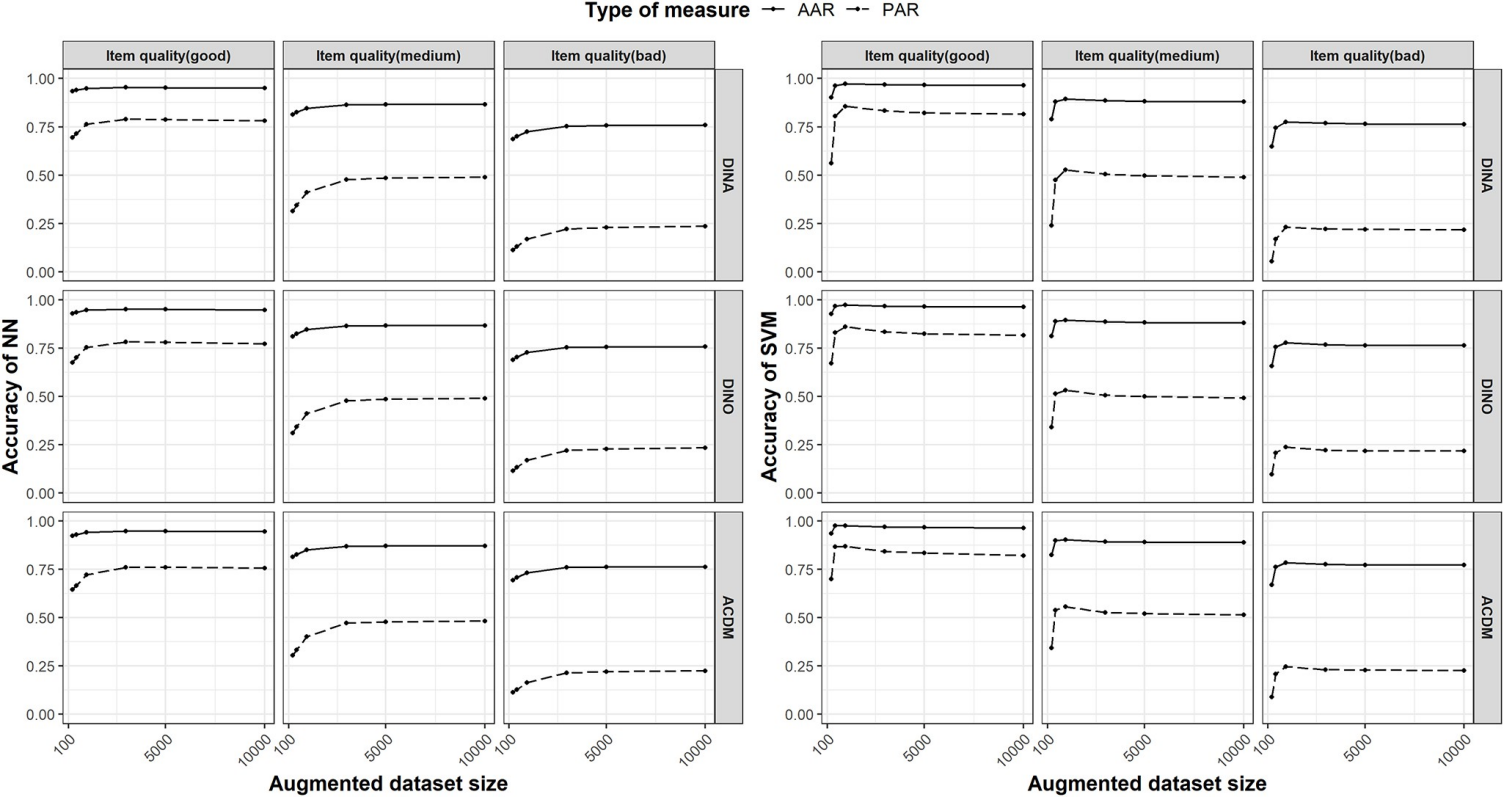
Effect of augmented dataset size on the accuracy of NN (left) and SVM (right). The solid line represents the pattern-wise agreement rate, while the dashed line indicates the attribute-wise agreement rate. The subgraphs in the first row illustrate the case when the item response generation model is DINA, while the second and third rows represent the cases for ACDM and DINO, respectively.

In the previous section, we evaluated the performance of NN and SVM models for SDCM-DA using an augmented dataset size *N* = 10,000, which was deemed sufficient. In this section, we explore the impact of the augmented dataset size on performance by comparing the results for *N* = 300, 500, 1,000, 3,000, 5,000, and 10,000. The PARs and AARs are averaged over six distinct error rates and six sizes of expert-labeled datasets, ensuring a comprehensive interpretation by factoring in multiple permutations. Fig 2 presents the PARs and AARs of NN and SVM with respect to the augmented dataset size under different item quality levels and item response models. As the size of the augmented dataset size *N* expands, the diagnostic accuracy of NN sees a gradual increase, with a slow convergence occurring from *N* = 1,000 to *N* = 10,000. Similarly, the accuracy of SVMs escalates with an increasing N, reaching its peak at N = 1,000. Beyond this point, the accuracy of SVMs either maintains a similar level or undergoes a slight decrease. Consequently, we advise that the size of the augmented dataset be set to a minimum of 1,000 when employing NN and SVM models in conjunction with the SDCM-DA method.

#### 4.5.3 Comparison to DCM with a condensation rule

**Fig 3:**
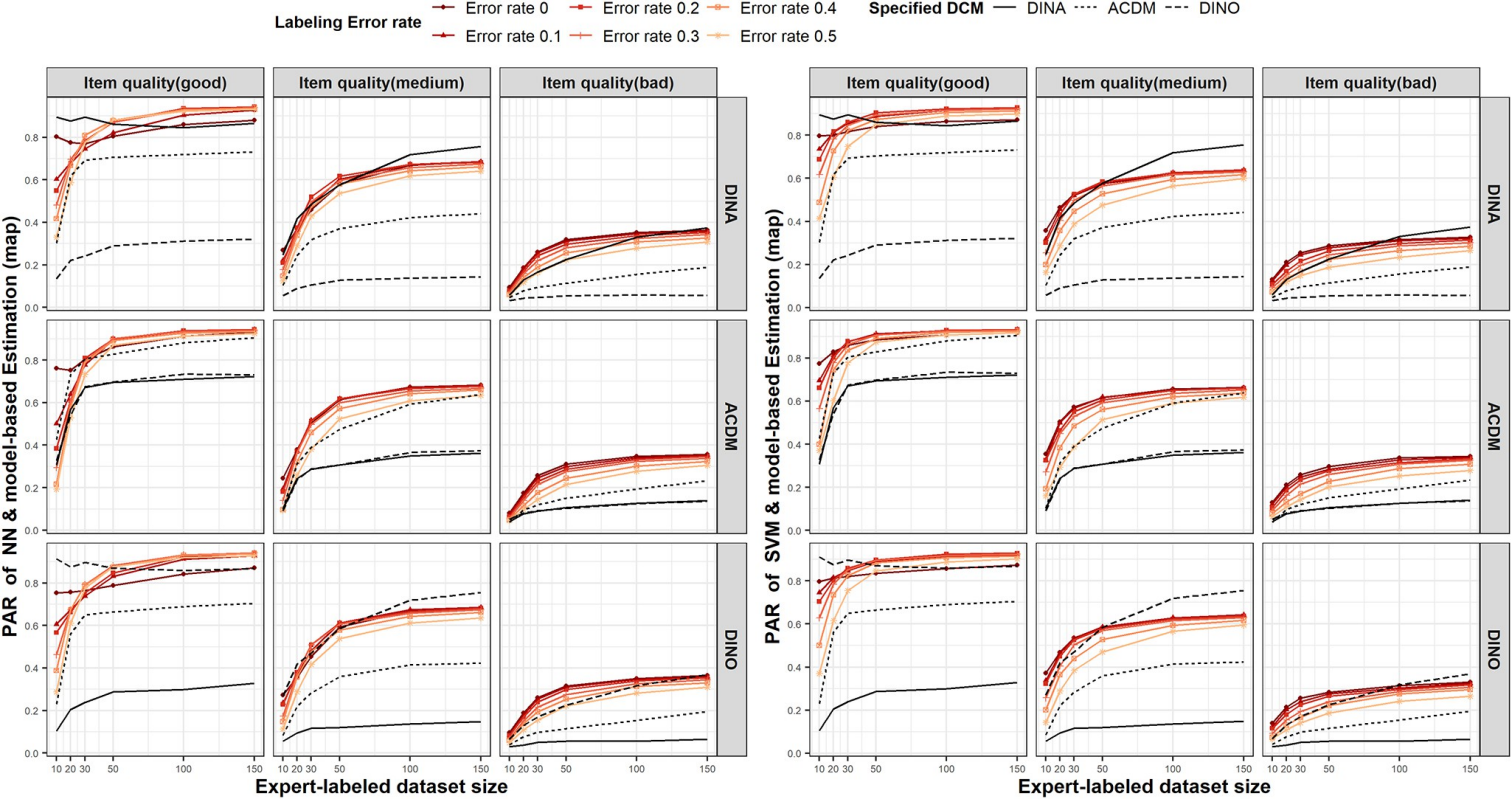
Pattern-wise agreement rates of augmentation method, NN (left) and SVM (right), and model-based estimation (map). The group of red solid lines represents the performance of models trained on the augmented training dataset. The degree of expert-labeling error rate is symbolized by the vividness of the colored lines, with a more vivid color indicating a smaller error rate. Additionally, the three black lines each represent the accuracy achieved when using model-based estimation, specifically by specifying the underlying item response model as either DINA, ACDM, or DINO.

The performance of the proposed SDCM-DA method was also pitted against the traditional model-based estimation approach, a method frequently applied in DCM. Fig 3 shows the results of the model-based estimation approach using map method alongside those of the SDCM-DA method, presented in Fig 1, for comparison. The exact numerical values for the model-based estimation approach, which includes the use of eap or mle methods and is substantially similar to the map method, are detailed in the result table of the S3 Appendix. Note that when the expert-labeled dataset size is too small, such as 10 and 20, the EM algorithm in CDM package in R fails to converge in approximately 11% and 1% of cases, respectively. In such cases, we regenerated the dataset under the same conditions until convergence was achieved, allowing us to report the results. This observation aligns with the criticism that DCM has limited utility for small samples [61–63].

The subgraphs in the first row of Fig 3 represent the diagnostic results obtained for the item response generation model of DINA. The second and third rows are for the item response generation model of ACDM and DINO, respectively. As anticipated, the diagnosis through model-based estimation delivers optimal performance when the true condensation rule of the item response generation model is accurately assumed. However, it presents significantly decreased performance when the model assumption is incorrect. For instance, when the genuine model is DINA, the diagnostic accuracy (PAR) plunges to just 0.32 if a DINO model is wrongly assumed for the simulation condition of high item quality and labeled dataset size of 150. This accuracy level is merely 37% of the accuracy (PAR = 0.87) that has achieved when the true DINA model is accurately employed. Similarly, when ACDM is incorrectly assumed as the model, the accuracy (PAR) drops to 0.73, which is 85% of the accuracy achieved by the correctly specified DINA model.

Typically, the model-free approach does not perform as well as the model-based approach that assumes a true model. Nevertheless, the model-assumption independent SDCM-DA method exhibits commendable diagnostic accuracy, recording PARs of 0.93 for NN and 0.90 for SVM, even with a high error rate of 0.5. Given that both NN and SVM, when trained solely on the expert-labeled dataset, demonstrated subpar performance in the presence of error rates, it indicates that the enhanced performance can be attributed to the data augmentation in SDCM-DA. The performance of SDCM-DA is either on par with or exceeds that of model-based estimation diagnoses under the correct model. Notably, it greatly outperforms diagnoses based on incorrectly specified condensation rules, even amidst labeling errors. Thus, in real-world applications, where identifying the accurate model can be challenging, the SDCM-DA presents itself as a promising alternative.

## 5 Discussion

This study proposed the SDCM-DA method that generates a sufficiently large training dataset from the correct response probabilities extracted from a small dataset with expert labels, and applies this training dataset to supervised learning for cognitive diagnosis. A simulation study was conducted taking into consideration five factors—item response models, item quality, the size of expert-labeled datasets, labeling error rate, and the size of augmented datasets—to evaluate the improved performance of SDCM-DA.

The results of the simulation study indicate that using the current SDCM-DA method significantly improves the diagnostic classification performance of compared to training on the expert-labeled datasets of size 150 or less. For NN, even with an error rate of 0.5 on a test composed of good-quality items, when the expert-labeled dataset of size 150, the simple augmentation method results in a PAR of 0.93, which is more than five times better than the PAR of 0.17 achieved by a classifier trained only with the expert-labeled dataset.

The improvement in classification performance through the augmented training dataset can be attributed to two primary factors. First, an augmented dataset contains data for all classes, unlike an expert-labeled dataset. Expert-label datasets often fail to include all classes due to their small sample size, and even the classes they do include only contain a very limited number of data points. For example, in a scenario diagnosing mastery of six attributes, with 2^6^ potential classes, expert-labeled datasets of sizes 10, 20, 30, 50, 100, and 150, regardless of the error rate, would only contain data points from an average of 9, 17, 24, 35, 51, and 58 classes, respectively, with only 0–3 data points per class. This could be the reason why, when training with only the expert-labeled dataset, performance varies so much depending on its size.

Secondly, the SDCM-DA method can serve as a promising approach to mitigate the impact of labeling errors. This method essentially converts expert labeling errors into item response errors through probabilities of correct answers. The considerable impact of labeling errors on classification performance is well-recognized [64], and studies systematically evaluating and comparing the effects of both labeling errors and response errors have demonstrated that labeling errors can be more detrimental to classification performance than response errors [65]. Please note that while the term ‘attribute error’ is used instead of ‘response error’ in [65], it carries a different connotation from ‘attribute’ as used in the current study. Consequently, classification trained on datasets generated using the SDCM-DA method, albeit imbued with response errors, can potentially demonstrate improved performance compared to training directly on an expert-labeled dataset that carries class errors.

In addition, our findings demonstrate that the SDCM-DA method, which eliminates the need for explicit model specification, delivers performance on par with the traditional model-based approach when the model is correctly specified, and outperforms when the model is inaccurately specified. The results suggest that the SDCM-DA approach is capable of generating effective augmented learning data that can successfully model real-world responses without the need to specify an underlying model. This feature, we believe, lends it significant potential for practical applications where a clear, predefined model may not always be available. Thus, the results suggest a path towards enhanced and more flexible methodologies in the realm of diagnostic classification.

This study represents a pioneering examination of data augmentation methods within the context of supervised learning applications in cognitive diagnosis, thereby revealing numerous aspects that require further exploration. A principal finding from our research suggests that data augmentation can significantly enhance the performance of diagnostic classification. However, it is crucial to understand that data augmentation does not always guarantee consistent performance improvements. Indeed, some studies have shown that an excessive increase in the amount of augmented data, when sufficient real-world data is already available, has the potential to degrade performance [21].

Building on this, our simulation study investigated the influence of the size of the augmented dataset, but it’s worth noting that the optimal size of the augmented training dataset may vary, being contingent on several factors including the number of attributes, the quantity of test items, and various factors related to noise or misspecification. Therefore, in practical applications, determining the optimal size of the augmentation to ensure maximum improvement should involve comparative analysis across various sizes of augmented data.

In relation to our study’s parameters, we adhered to the settings of previous research [17] by adopting a linear kernel for SVM, and in accordance with [15], we utilized a neural network with a single hidden layer of nine nodes. Alterations in these machine model configurations could potentially further enhance the performance of the NN or SVM models. However, the primary objective of this study was not to identify the most suitable models for each specific condition, but rather to propose, validate and document the appropriate data augmentation method capable of improving classification accuracy, particularly in scenarios with small item-response data with expert-labeling. Consequently, a comprehensive search for optimal models was beyond our current scope, but remains a subject of future research.

Moreover, it is noteworthy that our augmentation method involves random sampling the probabilities of a correct answer when the sparsity of the data prevents us from directly deriving the probabilities from the small data. This highlights a research need for techniques that permit thoroughly data-driven augmentation without such random sampling or the introduction of rules.

Finally, it should be acknowledged that the benefits of the SDCM-DA method extend beyond its application with Neural Networks (NNs) or Support Vector Machines (SVMs). Therefore, it is incumbent upon future research to assess the advantages that the SDCM-DA method provides across a more comprehensive range of supervised learning methods.

## Supporting information

S1 Appendix (DOCX)

S2 Appendix Result table of SDCM-DA. (XLSX)

S3 Appendix Result table of model-based estimation in DCMs. (XLSX)
