# Supplementary material for: Supervised diagnostic classification of cognitive attributes using data augmentation
Source: PLoS One. 2024 Jan 5;19(1):e0296464. doi: 10.1371/journal.pone.0296464 (PMC10769086; doi:10.1371/journal.pone.0296464)
Supplement: S1 Appendix — (DOCX) [file pone.0296464.s001.docx]

Table A. Q-matrix utilized in the simulation study

| Item | Attribute | | | | | | |
| --- | --- | --- | --- | --- | --- | --- | --- |
|  | $\alpha_{1}$ | $\alpha_{2}$ | $\alpha_{3}$ | $\alpha_{4}$ | $\alpha_{5}$ | $\alpha_{6}$ |  |
| 1 | 1 | 0 | 0 | 0 | 0 | 0 |  |
| 2 | 0 | 1 | 0 | 0 | 0 | 0 |  |
| 3 | 0 | 0 | 1 | 0 | 0 | 0 |  |
| 4 | 0 | 0 | 0 | 1 | 0 | 0 |  |
| 5 | 0 | 0 | 0 | 0 | 1 | 0 |  |
| 6 | 0 | 0 | 0 | 0 | 0 | 1 |  |
| 7 | 1 | 0 | 0 | 0 | 0 | 0 |  |
| 8 | 0 | 1 | 0 | 0 | 0 | 0 |  |
| 9 | 0 | 0 | 1 | 0 | 0 | 0 |  |
| 10 | 0 | 0 | 0 | 1 | 0 | 0 |  |
| 11 | 0 | 0 | 0 | 0 | 1 | 0 |  |
| 12 | 0 | 0 | 0 | 0 | 0 | 1 |  |
| 13 | 1 | 1 | 0 | 0 | 0 | 0 |  |
| 14 | 1 | 0 | 1 | 0 | 0 | 0 |  |
| 15 | 0 | 1 | 1 | 0 | 0 | 0 |  |
| 16 | 1 | 0 | 0 | 1 | 0 | 0 |  |
| 17 | 0 | 1 | 0 | 1 | 0 | 0 |  |
| 18 | 0 | 0 | 1 | 1 | 0 | 0 |  |
| 19 | 1 | 0 | 0 | 0 | 1 | 0 |  |
| 20 | 0 | 1 | 0 | 0 | 1 | 0 |  |
| 21 | 0 | 0 | 1 | 0 | 1 | 0 |  |
| 22 | 0 | 0 | 0 | 1 | 1 | 0 |  |
| 23 | 1 | 0 | 0 | 0 | 0 | 1 |  |
| 24 | 0 | 1 | 0 | 0 | 0 | 1 |  |
| 25 | 0 | 0 | 1 | 0 | 0 | 1 |  |
| 26 | 0 | 0 | 0 | 1 | 0 | 1 |  |
| 27 | 0 | 0 | 0 | 0 | 1 | 1 |  |
| 28 | 0 | 1 | 0 | 0 | 0 | 1 |  |
| 29 | 1 | 0 | 0 | 1 | 0 | 0 |  |
| 30 | 0 | 0 | 1 | 0 | 1 | 0 |  |

~~
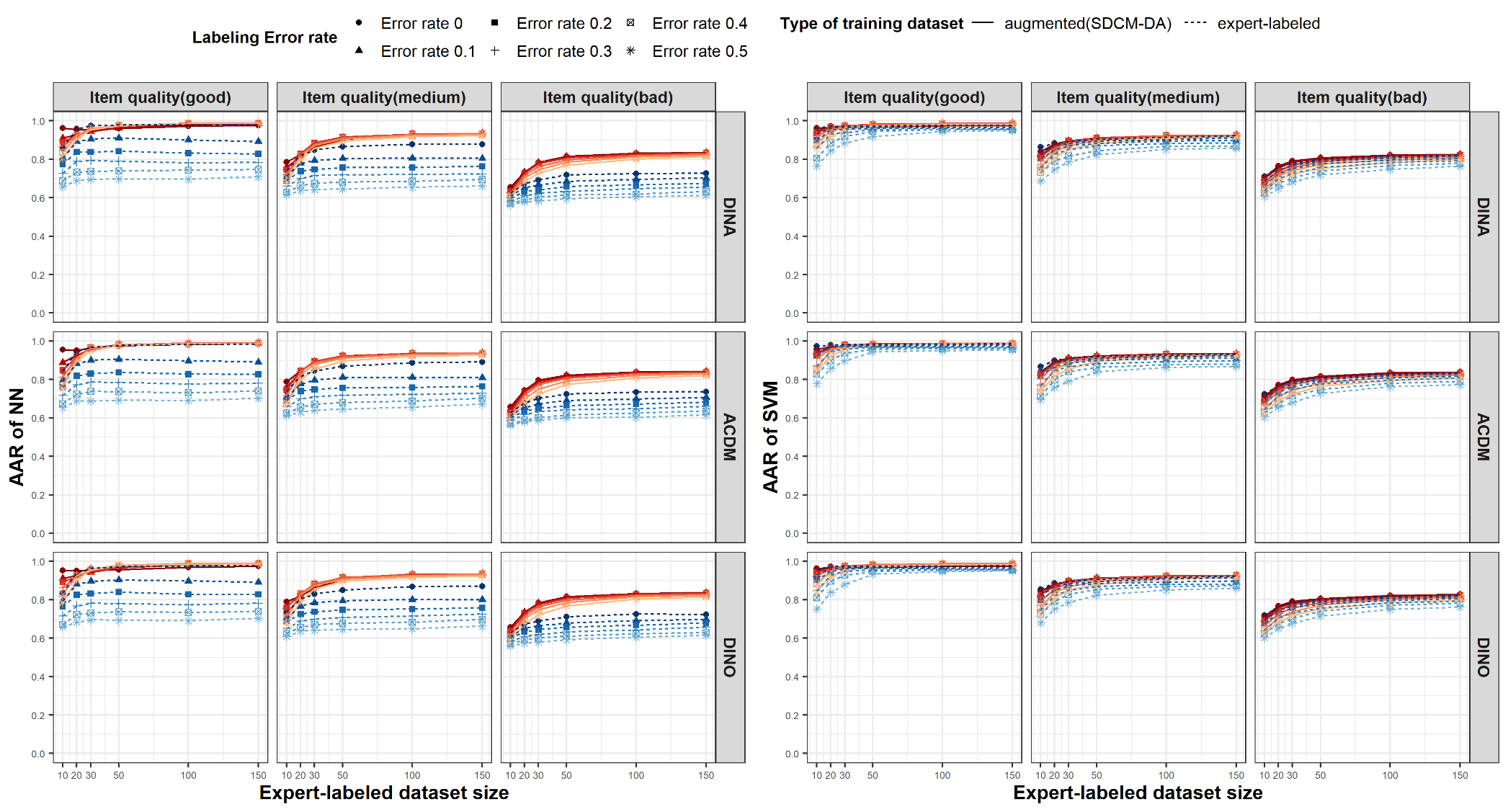
~~

Fig B. Attribute-wise agreement rates of NN (left) and SVM (right). The red solid line group represents the performance trained on the augmented training dataset, and the blue dashed line group represents the performance trained on the expert-labeled training dataset. The degree of expert-labeling error rate is indicated by the vividness of colored lines in each colored line group, with a more vivid color indicating a smaller error rate.

~~
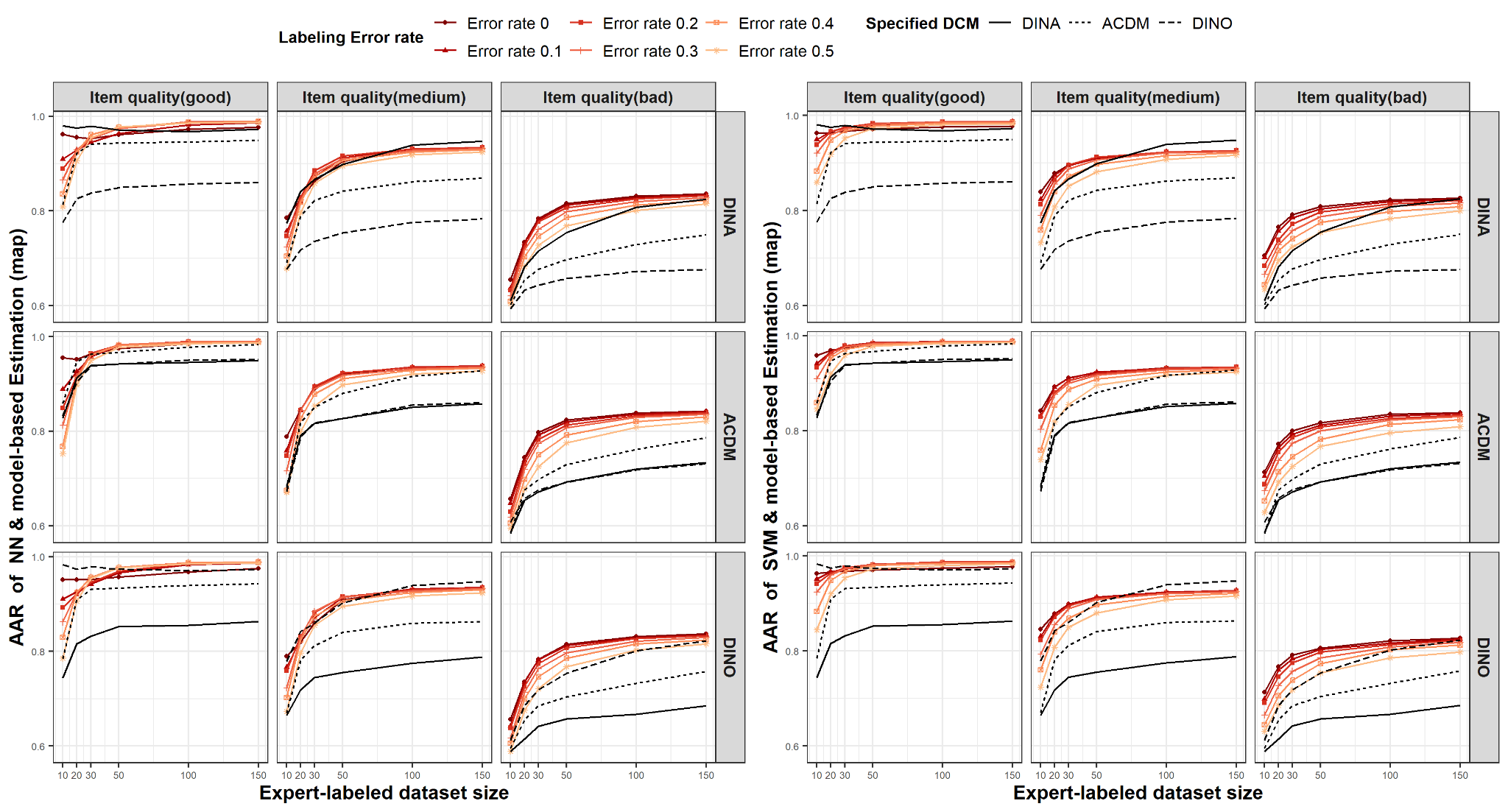
~~

Fig C. Attribute-wise agreement rates of augmentation method, NN (left) and SVM (right), and model-based estimation (map). The group of red solid lines represents the performance of models trained on the augmented training dataset. The degree of expert-labeling error rate is symbolized by the vividness of the colored lines, with a more vivid color indicating a smaller error rate. Additionally, the three black lines each represent the accuracy achieved when using model-based estimation, specifically by specifying the underlying item response model as either DINA, ACDM, or DINO.
